# Utilizing the Glucose and Insulin Response Shape of an Oral Glucose Tolerance Test to Predict Dysglycemia in Children with Overweight and Obesity, Ages 8–18 Years

**DOI:** 10.3390/diabetology5010008

**Published:** 2024-03-01

**Authors:** Timothy J. Renier, Htun Ja Mai, Zheshi Zheng, Mary Ellen Vajravelu, Emily Hirschfeld, Diane Gilbert-Diamond, Joyce M. Lee, Jennifer L. Meijer

**Affiliations:** 1 Department of Epidemiology, Geisel School of Medicine at Dartmouth, Hanover, NH 03755, USA;; 2 Department of Biostatistics, School of Public Health, University of Michigan, Ann Arbor, MI 48109, USA;; 3 Division of Pediatric Endocrinology, Diabetes and Metabolism, UPMC—Children’s Hospital of Pittsburgh, Pittsburgh, PA 15224, USA;; 4 Department of Pediatrics, Division of Pediatric Endocrinology, Susan B. Meister Child Health Evaluation and Research Center, University of Michigan, Ann Arbor, MI 48109, USA;; 5 Department of Medicine, Geisel School of Medicine at Dartmouth, Hanover, NH 03755, USA; 6 Department of Pediatrics, Geisel School of Medicine at Dartmouth, Hanover, NH 03755, USA; 7 Department of Medicine, Dartmouth-Hitchcock Medical Center, Lebanon, NH 03756, USA

**Keywords:** oral glucose tolerance test, insulin, glucose, curve shape, functional data analysis, pediatrics, hemoglobin A1C, longitudinal prediction, prediabetes

## Abstract

Common dysglycemia measurements including fasting plasma glucose (FPG), oral glucose tolerance test (OGTT)-derived 2 h plasma glucose, and hemoglobin A1c (HbA1c) have limitations for children. Dynamic OGTT glucose and insulin responses may better reflect underlying physiology. This analysis assessed glucose and insulin curve shapes utilizing classifications—biphasic, monophasic, or monotonically increasing—and functional principal components (FPCs) to predict future dysglycemia. The prospective cohort included 671 participants with no previous diabetes diagnosis (BMI percentile ≥ 85th, 8–18 years old); 193 returned for follow-up (median 14.5 months). Blood was collected every 30 min during the 2 h OGTT. Functional data analysis was performed on curves summarizing glucose and insulin responses. FPCs described variation in curve height (FPC1), time of peak (FPC2), and oscillation (FPC3). At baseline, both glucose and insulin FPC1 were significantly correlated with BMI percentile (Spearman correlation r = 0.22 and 0.48), triglycerides (r = 0.30 and 0.39), and HbA1c (r = 0.25 and 0.17). In longitudinal logistic regression analyses, glucose and insulin FPCs predicted future dysglycemia (AUC = 0.80) better than shape classifications (AUC = 0.69), HbA1c (AUC = 0.72), or FPG (AUC = 0.50). Further research should evaluate the utility of FPCs to predict metabolic diseases.

## 1. Introduction

The incidence of youth-onset type 2 diabetes (T2D) continues to increase by 4.8% each year, related to a high and increasing prevalence of pediatric obesity [1–3]. In the United States, diabetes care is among the highest health care expenditures, increasing by 26% from 2012 to 2017, further emphasizing the need for early detection of risk and prevention in youth [4]. The American Diabetes Association (ADA) recommends T2D screening using fasting plasma glucose (FPG), hemoglobin A1c (HbA1c), or a 2 h plasma glucose (2hrPG) during an oral glucose tolerance test (OGTT) [5,6]. During an OGTT, individuals consume a 75 g bolus of glucose after an overnight fast. The 2 h plasma glucose (2hrPG) level is indicative of normal glycemia, prediabetes, or T2D, with higher levels suggesting diminished beta cell response and/or decreased insulin sensitivity. However, cut-offs for FPG, HbA1c, and 2hrPG are crude estimates of dysglycemia [7], with uncertain implications for pediatric patients [8]. Recent studies have explored derived variables from insulin and glucose responses in an OGTT, including the sum of insulin across an OGTT [9] and one hour plasma glucose [9,10], which may better predict impaired glucose metabolism than HbA1c. Classifying the temporal response of glucose and insulin to an OGTT may provide a deeper understanding of metabolic risk than a single glucose or HbA1c measurement.

The pattern of glucose response to an OGTT reflects the ability of ß-cells to secrete insulin and the sensitivity of cells to lower glucose levels [11]. A study by Tschritter et al. [12] defined the shape of glucose in response to an OGTT as monophasic (one-peak of glucose), biphasic (second phasic insulin secretion), and incessant increase (continual rise of glucose, henceforth “monotonically increasing”) by collecting blood samples at baseline (0 min) and 30, 60, 90, and 120 min post-glucose bolus. In youth with obesity, a monotonically increasing glucose response is associated with the fastest deterioration of ß-cell function [13]. Furthermore, a monophasic glucose response is related to diminished ß-cell function, lower insulin sensitivity, and increased risk of metabolic syndrome in adults [14]. In a sample of adolescents with obesity, a biphasic glucose response was associated with the highest insulin sensitivity and lowest area under an OGTT insulin curve [15]. Another study found that adolescents with obesity who had a monophasic glucose response had lower insulin sensitivity and impaired β-cell function compared to those with a biphasic response, despite similar FPG and 2hrPG in the two groups [16]. Shape classifications are reasonably stable; a study of adults without a history of diabetes found that 59% maintained the same shape over three years, with either newly developing or maintaining a previous monophasic shape being associated with impaired glucose metabolism [17]. However, manually classifying the shape of glucose response to an OGTT is a relatively crude method of summarizing glucose response profiles, failing to account for multiple other ways in which profiles differ, such as the timing of the response peak and overall height [18,19].

Computational methods have been developed to systematically classify a curve shape, creating scores to optimally explain how participants vary in a study population. For instance, Frøslie et al. [20] used functional data analysis (FDA) to generate three functional principal components (FPCs) to classify the glycemic response to an OGTT among pregnant women in the first trimester (*n* = 974). They observed that the FPCs explained over 99% of variation in fitted OGTT curves, with the second FPC more accurately predicting gestational diabetes in the third trimester compared to traditional dysglycemia measures [20]. FDA methods have further been used to classify longitudinal trends of glucose, insulin, and blood pressure throughout pregnancy, identifying phenotypes through FDA that were associated with pregnancy-related outcomes [21]. Furthermore, FDA methods have been explored by Gecili et al. [22] for quantifying data from continuous glucose monitoring (CGM) in children with type 1 diabetes. They derived FPCs using data from CGMs to generate accurate real-time predictions of glycemic excursions. There have been no studies to our knowledge that have used computational FDA methods to classify the OGTT glucose and insulin response curve shape in youth without diabetes. Furthermore, there have been no studies that have compared computational methods with manual shape classifications and ADA classifications of dysglycemia to predict future dysglycemia in this population. The objectives of these analyses in youth with overweight and obesity were to evaluate cross-sectional associations of manual estimates of glucose response shape (monophasic, biphasic, and monotonically increasing) and a quantitative FDA method with markers of metabolic health, and to evaluate the longitudinal associations between these OGTT response characteristics and future dysglycemia.

## 2. Materials and Methods

### 2.1. Research Design

Participants in this analysis were a subset of 8–18-year-olds from a prospective cohort study designed to assess the longitudinal performance of tests for dysglycemia in children [23–26]. Participants were recruited through flyers, web postings, mailings, and research assistants at primary care and pediatric specialty clinics in southeast Michigan (2007–2019). Among previously documented exclusion criteria [24], participants were excluded if they had known diabetes or used medications known to affect the metabolism of glucose (oral steroids, metformin, insulin). For this analysis, all participants had overweight or obesity at baseline, defined by ≥85th percentile BMI from CDC 2000 growth charts [27]. Written informed consent was obtained from the parent/guardian for all participants. Written assent was obtained from participants ≥ 10 years old and verbal assent was obtained from participants < 10 years old. This study was approved by the University of Michigan Institutional Review Board (HUM#00006955).

Participants attended study visits at the Michigan Clinical Research Unit, where a medical history, vital signs, anthropometrics, and laboratory evaluation were performed. Our cohort represents a “convenience sample” with variations in the number of visits completed due to several grant mechanisms supporting different study aims. All participants attended an initial study visit, “Visit 1”, as previously documented and henceforth coined “baseline” [24]. All participants arrived at Visit 1 after an overnight fast (12 h). At Visit 1, participants underwent a 2 h OGTT with plasma glucose and insulin measurements; lab testing for HbA1c and lipids; blood pressure (measured twice with a pediatric cuff); measurement of height (twice), weight (twice, wearing a hospital gown), and body mass index (BMI) percentile per CDC growth curves [28]; waist circumference; and provided demographic information. Of 679 total participants with Visit 1 data, we excluded participants with incomplete OGTT glucose and insulin measurements (*n* = 7) and a missing HbA1c lab (*n* = 1), for a total cross-sectional analysis sample of *N* = 671.

Among this sample, a subset of the participants (*n* = 333) were recruited for longitudinal visits, supported by R01HD074559. This subset of participants completed two baseline OGTTs—Visit 1 (after an overnight fast) and Visit 2 (random fasted or fed state)—and two follow-up fasted OGTTs—Visit 3 and Visit 4—that were <4 weeks apart to assess for reproducibility in OGTT response. More details regarding the cohort structure are represented by Vajravelu et al. [24]. For this analysis, the longitudinal sample consisted of participants who returned for a follow-up OGTT after an overnight fast (*n* = 218), “Visit 3”, as previously described and henceforth coined “follow-up” [24]. Those with <6 months between Visit 1 and Visit 3 were excluded (*n* = 25), for a total longitudinal analysis sample of *N* = 193.

### 2.2. The Oral Glucose Tolerance Test

#### 2.2.1. OGTT Administration and Laboratory Parameters

An oral glucose load of 1.75 g per kg of body weight was administered up to a maximum of 75 g (Glucola, Fisherbrand, Waltham, MA, USA). Serial blood draws were performed at 30 min intervals for two hours and glucose homeostasis assays were conducted by the Michigan Diabetes Research Center (Ann Arbor, MI, USA). A Randox rX Daytona Chemistry Analyzer (Randox Laboratories Limited, Crumlin, UK) measured lipids (total cholesterol with the cholesterol enzymatic end point method, triglycerides with the GPO-PAP method, and HDL and LDL with the two step-direct method) and glucose with the glucose hexokinase method. A double-antibody radioimmunoassay was used to measure insulin. Both glucose and insulin were measured from plasma. Derived summary variables included FPG (0 min glucose), 2hrPG (120 min glucose), and dysglycemia, defined as having FPG > 100 mg/dL or 2hrPG > 140 mg/dL. HbA1c was quantified with a Tosoh G7 HPLC Analyzer (Tosoh Biosciences Inc., San Francisco, CA, USA).

#### 2.2.2. Manual OGTT Shape Classifications

**Figure 1.**
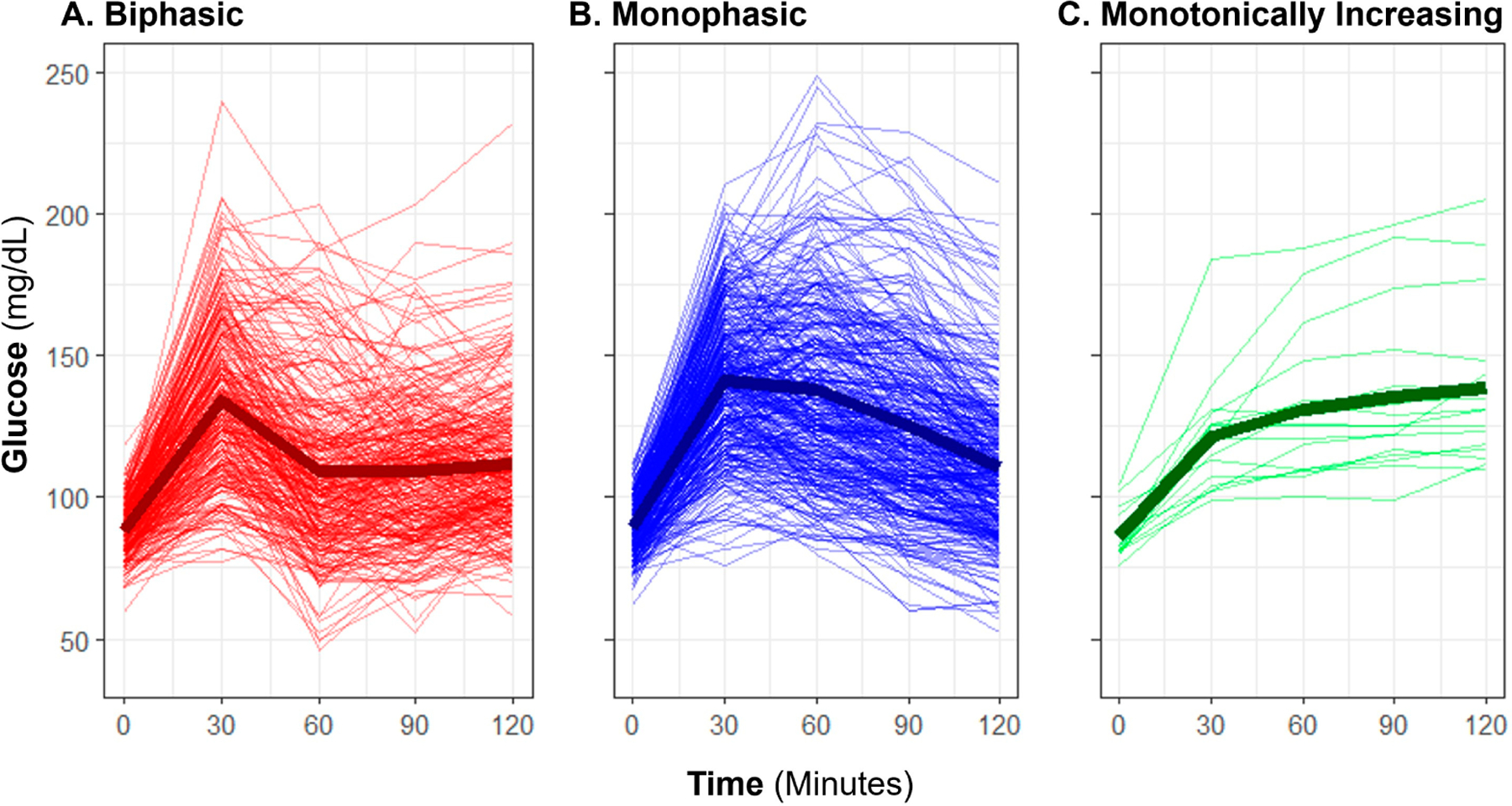
Observed oral glucose tolerance test biphasic, monophasic, and monotonically increasing glucose shape classifications. The plot displays profiles of glucose measurement for individuals (thin lines) and group means (bold lines) with each classification group: (**A**) biphasic, (**B**) monophasic, and (**C**) monotonically increasing.

Baseline OGTT profiles were manually classified as “biphasic,” “monophasic,” or “monotonically increasing” (previously also described as “incessant increase”) using criteria previously described in published literature [13,15,29]. A “biphasic” profile was defined by a rise in glucose over time with a subsequent decrease of at least 4.5 mg/dL from the initial peak, and a subsequent second increase of at least 4.5 mg/dL (Figure 1). A “monophasic” profile was defined by a rise in blood glucose that reached a peak with a subsequent decrease of at least 4.5 mg/dL from the maximum and no further increase exceeding 4.5 mg/dL. A “monotonically increasing” profile was defined as increasing blood glucose over time without a drop of more than 4.5 mg/dL from the maximum. An OGTT profile shape was considered “inconclusive” if there was no rise in glucose from baseline by 60 min. Manual shape classifications were only applied to glucose responses, not to insulin.

#### 2.2.3. OGTT Shape Classifications Using Functional Data Analysis (FDA)

The overall FDA procedure was previously described by Frøslie et al. [20]. Each participant’s five measurements of glucose and insulin were described by smooth curves in time. Then, FDA was applied to summarize each participant’s curve using a prefixed number of three principal component scores. The idea of FDA was to find the combination of basis functions that explain the most variance of the smooth curve and summarize them into the principal components. The implementation is briefly explained below.

The smooth curves were fitted with the *fda* package in R [30], using 7 basis functions and 5 measurements (knots). Thus, the glucose and insulin measurements could be viewed as a function of time, and formulized below:

(1) $$\text{glucos}e_{i}\left(t_{j}\right)=\overset{7}{\underset{k=1}{\sum }}{\phi_{k}}^{\text{glucose}}\left(t_{j}\right){c_{ik}}^{\text{glucose}}+{e_{i}}^{\text{glucose}}\left(t_{j}\right),i=1,\ldots ,n;j=1,\ldots ,5\;\text{insuli}n_{i}\left(t_{j}\right)=\overset{7}{\underset{k=1}{\sum }}{\phi_{k}}^{\text{insulin}}\left(t_{j}\right){c_{ik}}^{\text{insulin}}+{e_{i}}^{\text{insulin}}\left(t_{j}\right),i=1,\ldots ,n;j=1,\ldots ,5$$

where *t*_1_, …, *t*_5_ were the five measurement time points, *ϕ*_*k*_^*glucose*^(·), *ϕ*_*k*_^*insulin*^(·) were the k-th basis function for glucose and insulin, *c*_*ik*_^*glucose*^, *c*_*ik*_^*insulin*^ were the coefficients to be estimated for k-th basis function of i-th participant, and *e*_*i*_^*glucose*^(·), *e*_*i*_^*insulin*^(·) were the measurement error terms. The above formula could be simplified by using matrix notation:

(2) $$\text{Glucose}\;=\Phi^{\text{glucose}}C^{\text{glucose}}+E^{\text{glucose}};\;\text{Insulin}\;=\Phi^{\text{insulin}\;}C^{\text{insulin}\;}+E^{\text{insulin}\;}$$

where *Glucose*, *Insulin* were 5 × *n* matrices of glucose and insulin measurements, *Φ*^*glucose*^, *Φ^insulin^* were 5 × 7 matrices of basis functions taking value at the measurement time points, *C*^*glucose*^, *C*^*insulin*^ were 7 × *n* matrices of coefficients to be estimated, and *E*^*glucose*^, *E*^*insulin*^ were the measurement error matrices. Following the estimation methods described by Frøslie et al. [20], *C*^*glucose*^, *C*^*insulin*^ could be estimated with a penalized least squares method:

(3) $$C^{\text{glucose}}=\underset{C}{\text{argmin}}{\left(\text{Glucose}-\Phi^{\text{glucose}\;}C\right)}^{T}\left(\text{Glucose}-\Phi^{\text{glucose}\;}C\right)+\lambda_{1}C^{T}R_{1}C\;C^{\text{insulin}}=\underset{C}{\text{argmin}}{\left(\text{Insulin}-\Phi^{\text{insulin}\;}C\right)}^{T}\left(\text{Insulin}-\Phi^{\text{insulin}}C\right)+\lambda_{2}C^{T}R_{2}C$$

where the penalty term included *R*_1_, *R*_2_ that summarized the curvature of the curves, *λ*_1_, *λ*_2_ were nuisance parameters that control the penalty, and the nuisance parameters were determined by cross-validation [20].

After obtaining the smoothed curve, we used functional principal component analysis to summarize each participant’s curve into three scores. We fitted a series of FPC denoted as *ξ*_*k*_^*glucose*^(·), *ξ*_*k*_^*insulin*^(·) of the *k*-th component, such that they sequentially maximized the variance of the FPC scores ${z_{ik}}^{\text{glucose}}=\int {\xi_{k}}^{\text{glucose}}(t)\text{glucose}e_{i}(t)dt\;$ and ${z_{ik}}^{\text{insulin}}=\int {\xi_{k}}^{\text{insulin}}(t)\text{insuli}n_{i}(t)dt$ with constraints that $\int {\xi_{k}}^{\text{glucose}}{\left(t\right)}^{2}dt=1,\;\int {\xi_{k}}^{\text{insulin}}{\left(t\right)}^{2}dt=1$ and the glucose and insulin FPCs were orthogonal to each other separately. Following the choice made in Frøslie et al. [20], we chose to use the first three principal components for both glucose and insulin, and we found that the first three FPCs successfully explained over 99% of the variance for both glucose and insulin curves. We also standardized all three FPCs (mean = 0, standard deviation (SD) = 1) to enhance interpretability in statistical models.

### 2.3. Statistical Analysis

#### 2.3.1. Cross-Sectional Analysis

The summary statistics of participant demographics with respect to age, sex, race, and ethnicity were presented for the cross-sectional and longitudinal subsets, separately. Differences in demographics and measures of glucose regulation at baseline among those with and without follow-up were assessed with a Wilcoxon rank sum test (continuous variables) [31] or Fisher’s Exact Test (categorical variables) [32].

Classified by the three shaped glucose profiles “biphasic,” “monophasic,” or “monotonically increasing”, participants’ OGTT glucose profiles were plotted for the three sub-groups. Classified by quartiles of the top three FPC scores, participants’ OGTT glucose and insulin mean fitted curves were plotted and compared for the four sub-groups. The proportion of variance explained by the top three FPCs in glucose and insulin curves were calculated and reported. Using Spearman correlation coefficients, we also quantified the association of our classification measurements (manual shape classification and glucose/insulin FPC scores) with glucose and insulin measurements (at time point 0, 30, 60, 90 and 120 min), HbA1c, lipid labs, systolic and diastolic blood pressure, BMI percentile, and waist circumference. A Bonferroni correction was applied to adjust for multiple hypotheses in assessing numerous correlations (*p* < 0.0003, 171 comparisons). Differences in age, sex, race, ethnicity, and metabolic health parameters by manual OGTT shape classifications were assessed with Kruskal–Wallis’ Test [31] or Fisher’s Exact Test [32]. Linear regression evaluated associations between demographic variables with glucose and insulin FPC scores. To assess if associations between metabolic health parameters and FPCs were independent of demographic factors, we fit linear regression models for each FPC (dependent variable) by metabolic health parameters (independent variable), adjusting for demographic covariates.

#### 2.3.2. Longitudinal Analysis

Differences in FPG, 2hrPG, HbA1c, and BMI percentile between baseline and follow-up visits were assessed with paired Wilcoxon signed rank tests [31]. The difference in the rate of dysglycemia was assessed with McNemar’s test [32]. To assess the prediction ability of OGTT summary measures to predict future dysglycemia, a series of logistic regression models was used with dysglycemia at follow-up as the outcome variable and OGTT summary measures at baseline as predictors within the longitudinal subset. Seven different predictors or combinations of predictors were compared: FPG, 2hrPG, HbA1c, shape classifications, glucose FPCs, insulin FPCs, and glucose + insulin FPCs. Predictions for each participant’s probability of dysglycemia at follow-up from each model were used to generate receiver–operator curves (ROC), and the area under the ROC (AUC) was used to compare the performance of the predictors using the *pROC* R package [33]. All data analysis was conducted using the R Language for Statistical Computing [34].

## 3. Results

### 3.1. Sample Characteristics

Among the total cross-sectional analysis sample (*N* = 671), median age was 13.5 years (8.13 to 18.0 years), and 362 participants (53.9%) were female (Table 1). The sample was 42.5% non-white. Median BMI was at the 97th percentile, classified as having obesity.

### 3.2. Cross-Sectional Analysis

#### 3.2.1. Glucose Profile Manual Shape Classifications

The manual OGTT shape classifications yielded 42.0% of participants having the lowest risk associated [15,16] classification of “biphasic”, with a majority having “monophasic” shape (54.7%), and few with “monotonically increasing” (2.5%). For five participants (0.7%), the shape of the curve was inconclusive due to a lack of rise in glucose within the first hour in the test. The average profile for each shape subgroup is representative of the morphology expected for each (Figure 1); however, individual participant glucose responses varied widely in height and peak time within each classification.

#### 3.2.2. Glucose and Insulin FDA Shape Characteristics

**Figure 2.**
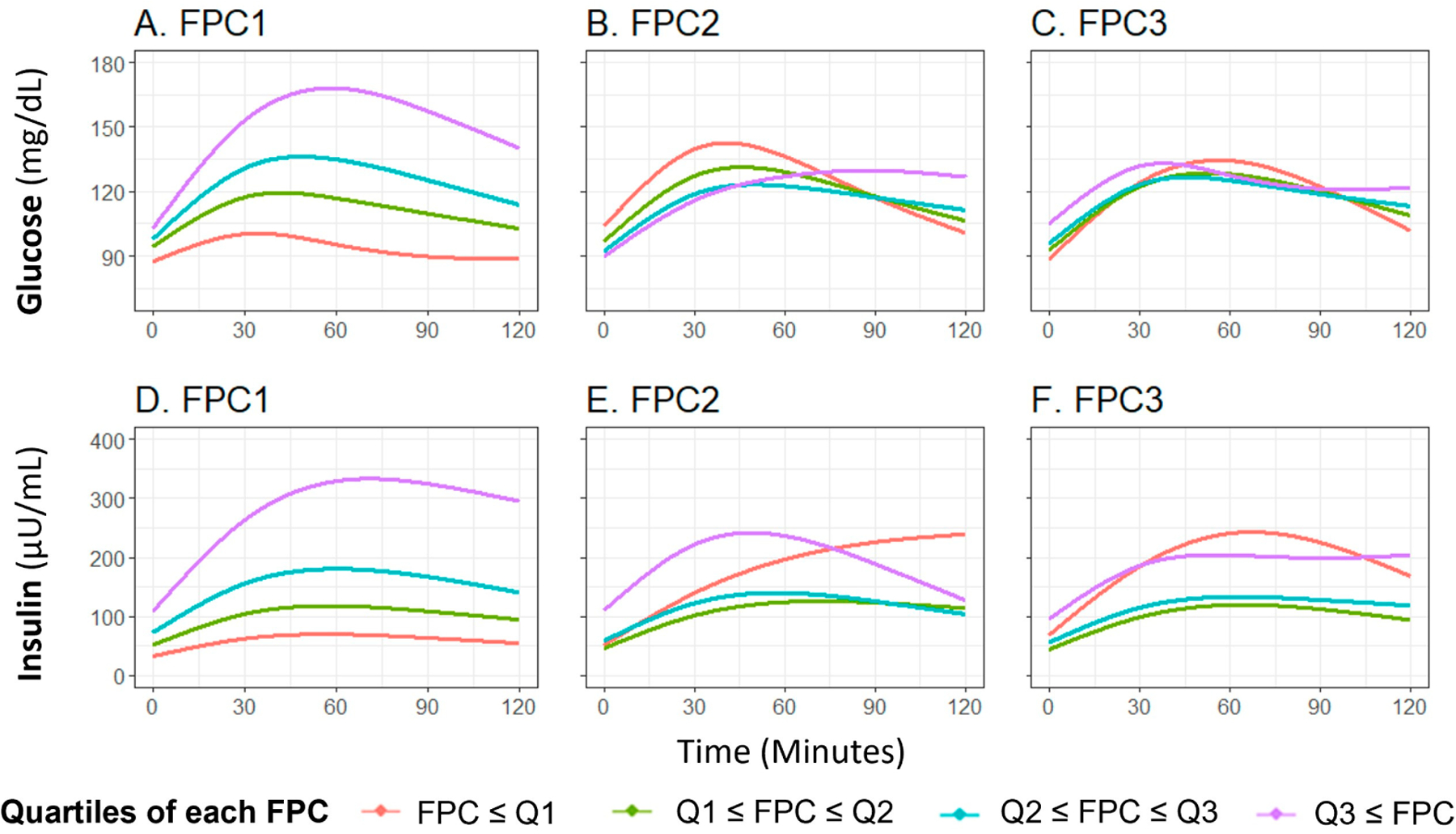
Means of fitted curves of the oral glucose tolerance test curves for glucose and insulin by FPC scores quartile classification. The plot shows average fitted curves of glucose (**A**–**C**) and insulin (**D**–**F**) responses, classified by the quartile of each FPC score ((**A**,**D**): FPC1 score; (**B**,**E**): FPC2 score, and (**C**,**F**): FPC3 score). Abbreviations: FPC, functional principal component.

The first three glucose FPCs explained 89.6%, 7.9%, and 2.1% of the variance in the glucose curves, respectively (Figure S1A–C). FPC1 score successfully characterized the height of the glucose curve, meaning participants with higher FPC1 score are more likely to have a higher glucose level throughout the OGTT (Figure 2A), FPC2 and FPC3 scores characterized the shape of the glucose curve, with FPC2 relating to the timing and height of the peak value (Figure 2B) and FPC3 relating to oscillation (Figure 2C). These shape characteristics are further demonstrated by plotting the glucose curves for participants with the highest and lowest decile of each FPC score (Figure S2A–C).

The first three insulin FPCs captured similar variation, with 92.2%, 7.0%, and 0.7% of variation in the insulin curves explained by the first three FPCs, respectively (Figure S1D–F). Similar to the findings with glucose, FPC1 score characterized the overall height of the insulin curve (Figure 2D), FPC2 score characterized the timing of peak (Figure 2E), and FPC3 score related to the oscillation (Figure 2F). The difference of insulin curves between participants with extreme high/low deciles of FPC scores are presented in Figure S2D,E.

#### 3.2.3. Cross-Sectional Associations with Metabolic Health Parameters

**Figure 3.**
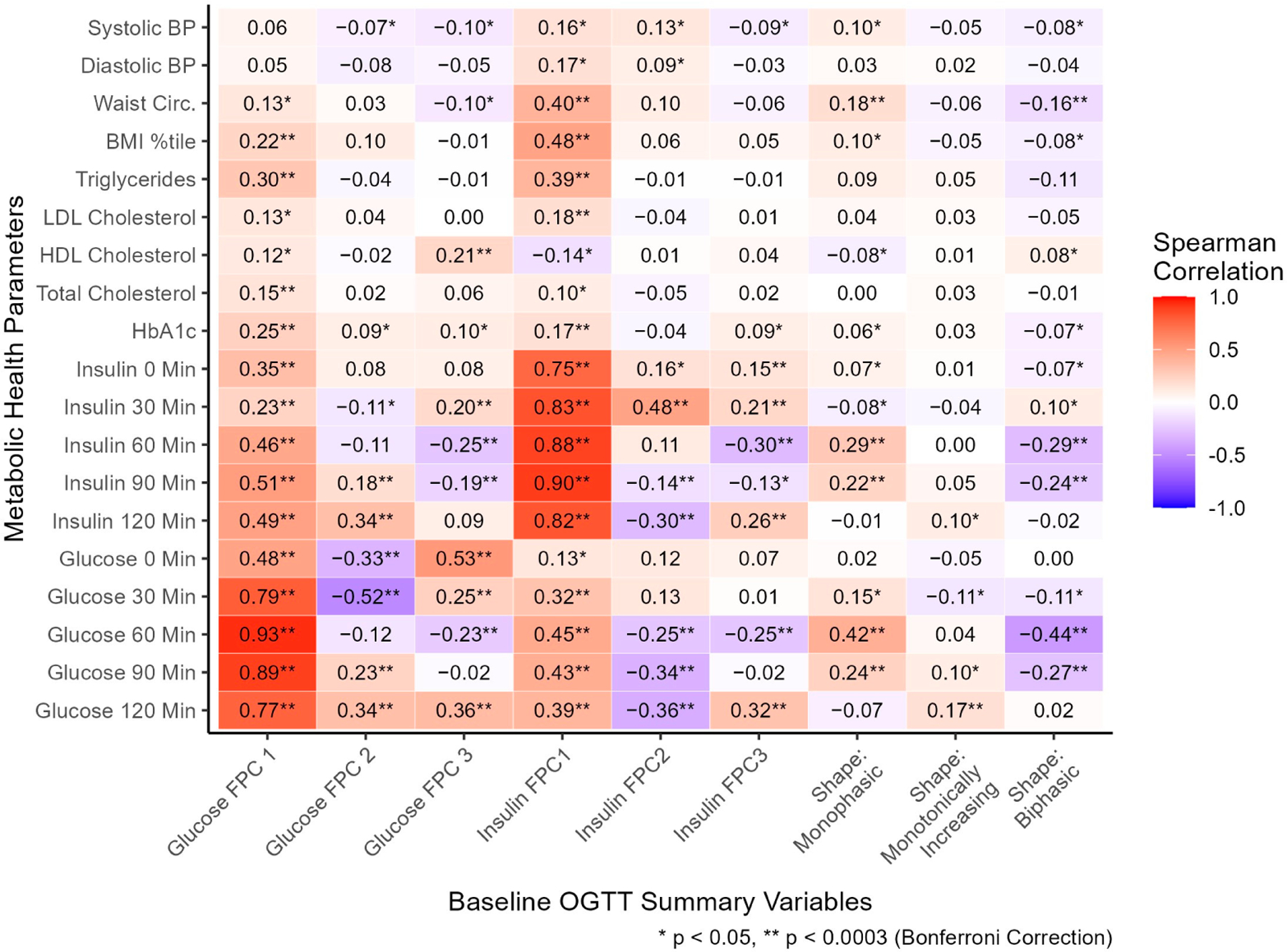
At baseline, the relationship between oral glucose tolerance test shape variables and metabolic health parameters. All FPC scores were standardized as continuous variables. Shape classifications were coded as separate dichotomous variables, with five participants excluded with unclassified shapes. Spearman correlations were utilized to assess associations. Units: systolic/diastolic BP (mmHg), waist circumference (cm), triglycerides/HDL/LDL/total cholesterol (mg/dL), HbA1c (%), insulin (μU/mL), glucose (mg/dL). Abbreviations: BP, blood pressure; BMI, body mass index; LDL, low-density lipoprotein; HDL, high-density lipoprotein; HbA1c, hemoglobin A1c; FPC, functional principal component. Variable-specific missingness existed for BP (*n* = 21), waist circumference (*n* = 24), and lipid labs (*n* = 10).

Glucose manual profile shapes (coded as dichotomous variables) and FPC scores summarizing glucose and insulin curve shapes (coded as continuous variables) were compared with metabolic health parameters, cross-sectionally (Figure 3). The monophasic shape was significantly associated with higher 60- (r = 0.42), and 90- (r = 0.24) minute glucose, monotonically increasing shape with higher 120 min glucose (r = 0.17), and biphasic with lower 60- (r = −0.44) and 90- (r = −0.27) minute glucose. The monophasic glucose shape was associated with higher 60- (r = 0.29) and 90- (r = 0.22) minute insulin, and biphasic with significantly lower 60- (r = −0.29) and 90- (r = −0.24) minute insulin. The directions of associations of glucose shape classifications with glucose and insulin values support the validity of these classifications to describe the general shape of responses. Although the shape classifications capture the general shape of intra-person response in plasma glucose, these moderate correlations highlight substantial response variability utilizing the manual shape classifications. Other metabolic health parameters were associated with manual shape classification (Figure 3, Table S1): waist circumference (WC) was positively associated with monophasic shape (r = 0.18, median monophasic WC = 93 cm) and negatively with biphasic shape (r = −0.16, median biphasic WC = 88 cm). The shape classifications differed significantly by age and ethnicity, but not by sex or race (Table S1).

Glucose FPC1 score was positively correlated with glucose at each time point throughout the OGTT (r = 0.48, 0.79, 0.93, 0.89, and 0.77; Figure 3), consistent with its general explanation of overall curve height. Glucose FPC2 score was negatively correlated with 0- (r = −0.33), and 30- (r = −0.52) minute glucose and positively correlated with 90- (r = 0.23) and 120- (r = 0.34) minute glucose, consistent with a general explanation of time to peak captured by FPC2 score. Glucose FPC3 score was positively correlated with 0- (r = 0.53), 30- (r = 0.25), and 120- (r = 0.36) minute glucose and negatively correlated with 60 min glucose (r = −0.23), consistent with its general explanation of oscillation in each curve. All three insulin FPC scores followed similar patterns of association with insulin at each time as the glucose FPC scores did with glucose at each time, supporting the characteristics qualitatively captured by each FPC. Furthermore, multiple significant associations were found between glucose FPCs and plasma insulin, and between insulin FPCs and plasma glucose, suggesting each type of FPC (glucose or insulin) is strongly related to levels of the other marker.

Glucose FPC1 score was significantly correlated with HbA1c (r = 0.25), total cholesterol (r = 0.15), triglycerides (r = 0.30), and BMI percentile (r = 0.22). Glucose FPC2 score was not significantly correlated with any metabolic health parameters. Glucose FPC3 score was significantly correlated with HDL cholesterol (r = 0.21). Insulin FPC1 score was significantly correlated with HbA1c (r = 0.17), LDL cholesterol (r = 0.18), triglycerides (r = 0.39), BMI percentile (r = 0.48), and waist circumference (r = 0.40). Insulin FPC2 and FPC3 scores were not significantly correlated with any metabolic health parameters after multiple hypothesis correction.

We evaluated associations of age, sex, race, and ethnicity (independent variable) with glucose and insulin FPC scores (dependent variables) in separate linear regression models. Several significant associations were found between these demographic factors and the FPC scores, as reported in Tables S2 (glucose FPC scores) and S3 (insulin FPC scores). Therefore, to evaluate associations between the metabolic health parameters (independent variables) and glucose and insulin FPC scores (dependent variables) independent of demographic factors, we fit linear regression models adjusting for age, sex, race, and ethnicity. As reported in Tables S4 (models for glucose FPC scores) and S5 (models for insulin FPC scores), numerous significant associations were found. Notably, these adjusted regression findings concurred with all significant correlations of metabolic health parameters and glucose and insulin FPC scores reported in Figure 3, suggesting these associations are independent of demographic factors.

### 3.3. Longitudinal Analysis

A total of 193 participants completed the follow-up assessment at least six months after baseline. Age at baseline, sex, and ethnicity did not differ significantly among participants who also had complete longitudinal data compared with those in the cross-sectional analysis alone, though there were significant differences in race and some baseline metabolic health parameters (Table S6). In the longitudinal subset, median follow-up time was 14.5 months (Table 2). Paired FPG (*p* < 0.001) and HbA1c (HbA1c) changed significantly between baseline and follow-up, though 2hrPG and BMI percentile did not. Though the overall rate of dysglycemia was 7.3% (*n* = 14) at both baseline and follow-up, just four participants with dysglycemia at baseline still had dysglycemia at follow-up (28.6%), meaning the same number of participants (*n* = 10) newly developed dysglycemia as those who no longer had it.

**Figure 4.**
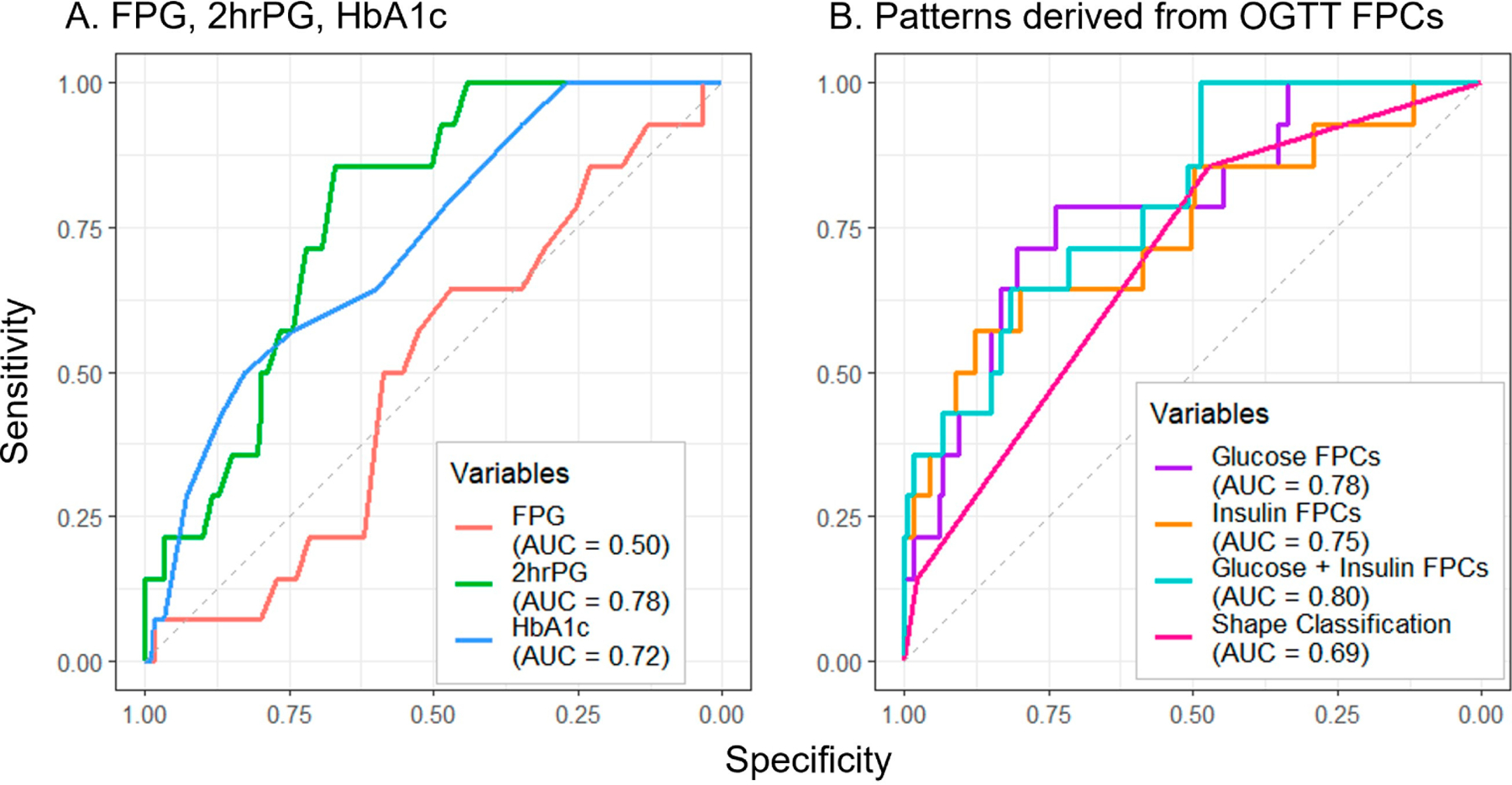
Utilizing baseline oral glucose tolerance test parameters to predict follow-up dysglycemia. Predictors include single laboratory values (**A**) and methods using OGTT response shape information (**B**). Abbreviations: FPG, fasting plasma glucose; 2hrPG, 2 h plasma glucose; HbA1c, hemoglobin A1c; OGTT, oral glucose tolerance test; FPC, functional principal component; AUC, area under the curve.

Of all measures assessed to predict future dysglycemia, FPG performed the poorest (AUC = 0.50) (Figure 4A). Though the manual shape classifications (AUC = 0.69) (Figure 4B) exceeded the performance of FPG, greater prediction accuracy was achieved by 2hrPG (AUC = 0.78) and HbA1c (AUC = 0.72) (Figure 4A). The combination of glucose and insulin FPCs yielded the greatest prediction accuracy (AUC = 0.80) (Figure 4B), with notable improvements in test sensitivity at higher values of specificity for all FPC approaches when compared to the other approaches.

## 4. Discussion

To our knowledge, this is the first study that has used FDA for summarizing OGTT glucose and insulin responses to evaluate the association of FPC scores with metabolic health markers and future dysglycemia in a diverse adolescent population. We sought to utilize FDA to characterize the glucose and insulin response to an OGTT because single measures and manual shape classifications may be missing information on the dynamic response to a glucose bolus. Glucose and insulin FPC scores had substantially stronger associations with metabolic health parameters than manual shape classifications, being significantly correlated with waist circumference, BMI percentile, LDL/HDL/total cholesterol, triglycerides, and HbA1c. Though differences in FPC scores by age, sex, and race were noted, these associations were still observed in models adjusting for demographic covariates. In longitudinal logistic regression analyses, glucose and insulin FPC scores predicted future dysglycemia better than manual shape classifications, HbA1c, or FPG.

Multiple characteristics of glucose and insulin curves were captured by the FPCs. Glucose and insulin FPC1 explained variation in overall curve height, FPC2 explained the positioning of the peak, and FPC3 explained oscillations in the curves. These attributes are similar to previous findings from use of this method in OGTTs from the first trimester of pregnancy, supporting this method as consistent across OGTTs for multiple applications [20]. Given this extent of variance explained, the FPC scores numerically captured some variation qualitatively related to the glucose shape classifications. Additional exploratory analysis revealed monophasic shape was positively correlated with FPC1 (r = 0.28) and negatively with FPC2 (r = −0.18) and FPC3 (r = −0.51) scores, indicating a high curve that peaks mid/late with little curvature. The biphasic shape was notably negatively correlated with FPC1 (r = −0.30), and positively with FPC3 (r = 0.50) scores, indicating a curve with a low overall level and high amount of oscillation. Monotonically increasing shape was notably positively associated with FPC2 score (r = 0.27), indicating a late peak.

Previously, manual glucose curve shape classifications have been associated with differences in β-cell function and insulin sensitivity in both children and adults [13,14,16]. Furthermore, measures of insulin in an OGTT may enhance the assessment of impaired glucose metabolism [9]. By using glucose and insulin FPCs, multiple modes of variation in both glucose and insulin are captured across time and may more comprehensively assess disordered glucose regulation and metabolism. To our knowledge, no other method captures this variation in both glucose and insulin. The performance of the FPC scores for predicting future dysglycemia supports their utility. Consistent with concerns about generalizing methods of classifying dysglycemia using single laboratory values to children [7,8], only 28.6% of children with dysglycemia at baseline (defined by a composite of elevated FPG or 2hrPG) still had it at follow-up. When predicting this longitudinal dysglycemia, the glucose and insulin FPC scores outperformed both single laboratory values (FPG, 2hrPG, HbA1c) and the manual shape classifications. Though for the application of gestational diabetes, this is consistent with a previous findings that glucose FPC2 score in the first trimester of pregnancy is a significant predictor of gestational diabetes in the third, while other glucose summary measures were not [20].

The sample was large, racially and ethnically diverse, and had a reasonably long median follow-up time over which to observe longitudinal changes in dysglycemia. These findings are therefore reasonably generalizable to children ages 8–18 with overweight or obesity and without a previous diagnosis of diabetes. Several limitations are important to note. First, the study cohort did not consistently collect physician-assessed tanner staging to determine pubertal status and puberty is associated with a physiologically normal decrease in insulin sensitivity [35]. However, obesity and metabolic disease are known to affect this physiologic change during puberty [35,36], so it is unclear how to best account for puberty in relation to fitting and using FPC scores. This analysis adjusted for age and sex in cross-sectional models to account for puberty-related differences. Additionally, few participants had dysglycemia at follow-up, preventing the split of the cohort into training and validation samples for longitudinal prediction. This low overall rate of dysglycemia at follow-up seems to be related to observed differential follow-up completion by health status, where “healthier” children were more likely to complete follow-up. The longitudinal predictive value of the FPC scores should be further confirmed in other cohorts.

Though the OGTT method used in this study required serial blood draws and would likely be burdensome to use as a clinical predictive screening test for otherwise healthy children, future work should validate and adapt the use of OGTT FPCs for a clinical setting. Further research is needed to translate individual glucose and insulin FPC scores to clinically useful classifications of risk, a potentially valuable tool to include in an electronic medical record. Additionally, previous studies have used similar functional data analysis methods with continuous glucose monitoring (CGM) data in type I diabetes [22]. A CGM-based screening tool could be developed to detect early abnormal glucose regulation for children without known diabetes; such a method would be minimally burdensome to the patient and could incorporate many more data points to better fit precise glucose curves.

## 5. Conclusions

This analysis suggested that beyond simple plasma glucose values, glucose and insulin curve shape information derived from OGTTs more directly profiles underlying physiology in a way that is meaningfully associated with metabolic health parameters and longitudinally with future dysglycemia in children with overweight or obesity. Glucose and insulin FPC scores from an OGTT present a powerful way to summarize this shape information. FPCs may be clinically useful to predict future dysglycemia among children at elevated risk due to their body weight, allowing for enhanced early intervention.

## Supplementary Material

diabetology-2784765-supplementary

## Figures and Tables

**Table 1.** Participant Characteristics at Baseline.

	Overall (*N* = 671)^1^
Study Visits Completed	
Baseline and Follow-Up	193 (28.8%)
Baseline Only	478 (71.2%)
Age (Years)	13.5 [11.5, 15.4]
Sex	
Female	362 (53.9%)
Male	309 (46.1%)
Race	
White	386 (57.5%)
Black or African American	212 (31.6%)
Other/Multiracial	52 (7.8%)
Unknown/Not Reported	21 (3.1%)
Ethnicity	
Non-Hispanic/Latino	632 (94.2%)
Hispanic/Latino	39 (5.8%)
BMI Percentile	97.0 [94.1, 98.8]
OGTT Curve Shape Classification	
Monophasic	367 (54.7%)
Biphasic	282 (42.0%)
Monotonically Increasing	17 (2.5%)
Inconclusive	5 (0.7%)

Abbreviations: BMI, body mass index; OGTT, oral glucose tolerance test; Q1, 1st quartile; Q3, 3rd quartile.

1 Median [Q1, Q3] or n (%).

**Table 2.** Comparison of characteristics of participants between baseline and follow-up from the longitudinal subset (*N* = 193).

Characteristic	At Baseline ^1^	At Follow-Up ^1^	*p*-Value^2^
Age (years)	13.3 [11.5, 15.3]	14.7 [12.9, 16.5]	
Δ Age (months)		14.5 [12.6, 17.2]	
Dysglycemia			1.000
No (%)	179 (92.7%)	179 (92.7%)	
Yes (%)	14 (7.3%)	14 (7.3%)	
FPG (mg/dL)	83 [79, 90]	87 [83, 91]	<0.001
2hrPG (mg/dL)	99 [86, 114]	102 [86, 114]	0.438
HbA1c (%)^3^	5.2 [5.0, 5.4]	5.2 [5.0, 5.4]	<0.001
BMI Percentile	96.2 [92.1, 98.6]	96.3 [91.5, 98.8]	0.567

Abbreviations: FPG, fasting plasma glucose; 2hrPG, 2 h plasma glucose; HbA1c, hemoglobin A1c; BMI, body mass index; Q1, 1st quartile; Q3, 3rd quartile.

1 Median [Q1, Q3] or n (%).

2 Paired Wilcoxon signed rank tests (continuous variables) or McNemar’s test (dysglycemia).

3 One participant had missing HbA1c at follow-up. Median change in HbA1c was 0.1 [−0.1, 0.2].

## Data Availability

The datasets presented in this article are not readily available due to privacy and IRB regulatory restrictions. Requests to access the datasets should be directed to the authors.
