# Supplementary material for: Utilizing the Glucose and Insulin Response Shape of an Oral Glucose Tolerance Test to Predict Dysglycemia in Children with Overweight and Obesity, Ages 8–18 Years
Source: Diabetology (Basel). Author manuscript; Available in PMC 2024 Apr 4. (PMC10994153; doi:10.3390/diabetology5010008)
Supplement: diabetology-2784765-supplementary [file NIHMS1978392-supplement-diabetology-2784765-supplementary.pdf]

Supplementary Tables and Figures

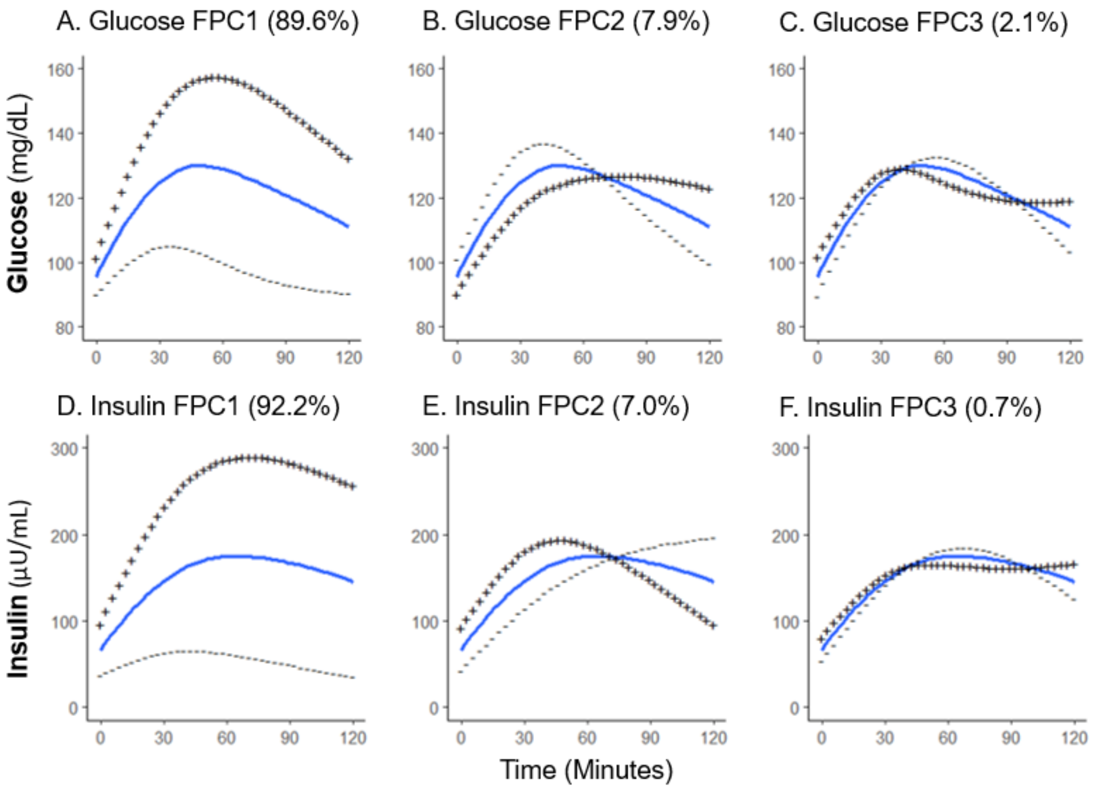

Figure S1. Fitted FPC curves for glucose and insulin (% variance explained). Fitted curves are shown for average glucose (A-C) and insulin (D-F) FPCs with +/- curves indicating the fitted curve for one standard deviation +/- the mean score for each FPC (error designation calculated as in Frøslie et al. [20]). Percent variance explained is the proportion of variance in fitted curves for each participant explained by each FPC. Abbreviations: FPC, functional principal component.

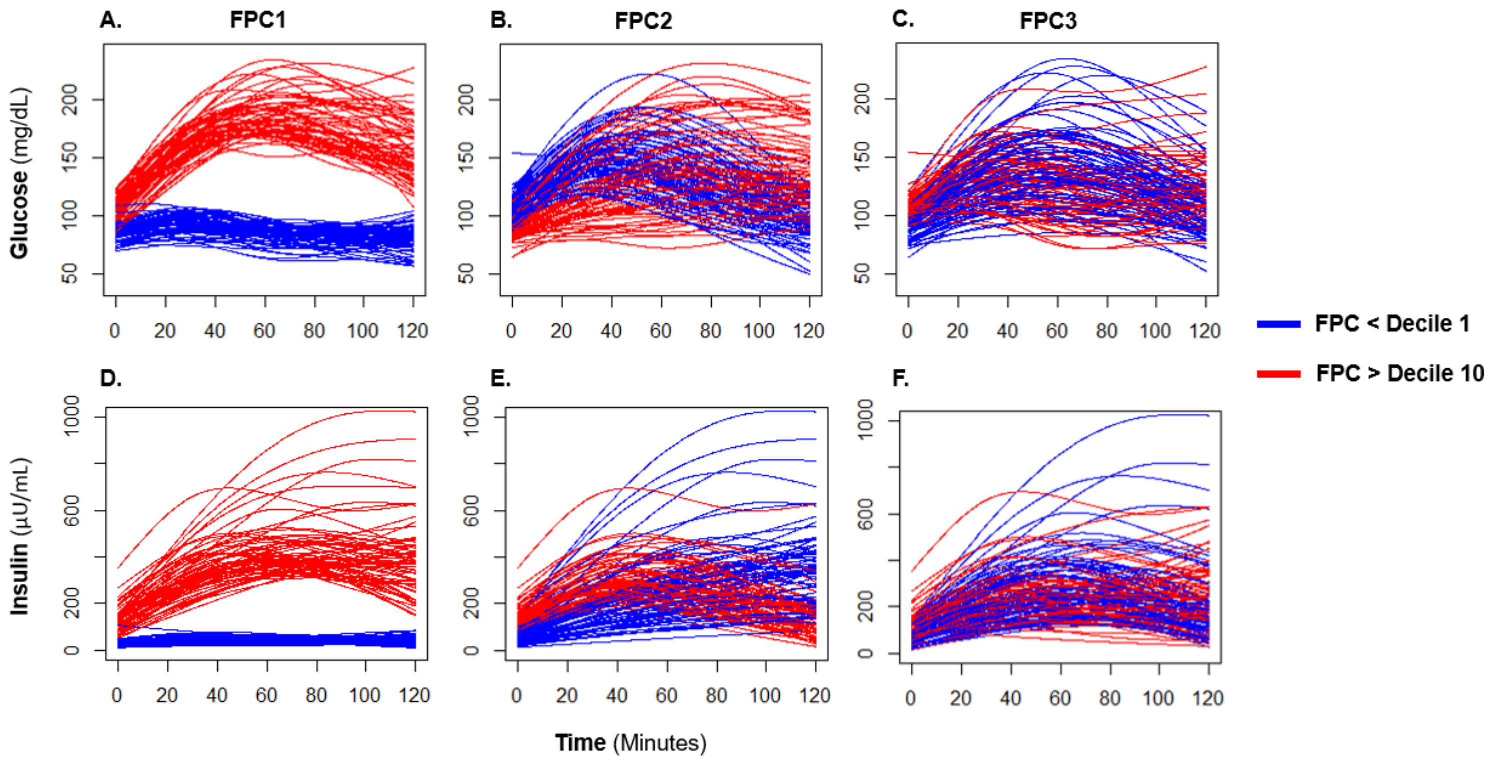

Figure S2. Curves from extreme deciles of each FPC. Fitted curves are shown from participants from extreme deciles of the three glucose (A-C) and insulin (D-F) FPCs. Participants with extreme values were selected separately for each FPC, so the subset of participants included in each plot is not the same. Abbreviations: FPC, functional principal component.

Table S1. Metabolic health parameters by shape classification (cross-sectional)

Characteristic	**Monophasic**, n = 368[1]	**Biphasic**, n = 282[1]	**Incessant Increase**, n = 17[1]	p-value[2]
**Age (Years)**	13.7 [12.1, 15.5]	12.8 [11.2, 15.0]	14.6 [11.7, 15.69]	**<0.001**
**Sex**				0.174
Female	188 (52%)	159 (44%)	12 (3.3%)	
Male	179 (58%)	123 (40%)	5 (1.6%)	
**Race**				0.272
White	225 (59%)	152 (40%)	7 (1.8%)	
Black or African American	107 (51%)	95 (45%)	7 (3.3%)	
Other/Multiracial	25 (48%)	25 (48%)	2 (3.8%)	
Unknown/Not Reported	10 (48%)	10 (48%)	1 (4.7%)	
**Ethnicity**				**0.034**
Non-Hispanic/Latino	342 (55%)	271 (43%)	14 (2.2%)	
Hispanic/Latino	25 (64%)	11 (28%)	3 (7.7%)	
**BMI Percentile**	97.3 [94.7, 98.9]	96.8 [93.4, 98.6]	96.6 [94.6, 97.8]	0.051
**Waist Circumference (cm)**	93 [85, 104]	88 [80, 98]	85 [81, 94]	**<0.001**
**Systolic BP (mmHg)**	115 [107, 124]	112 [106, 121]	113 [106, 115]	**0.037**
**Diastolic BP (mmHg)**	63 [59, 68]	62 [58, 67]	63 [60, 68]	0.567
**HbA1c (%)**	5.4 [5.1, 5.7]	5.3 [5.1, 5.6]	5.6 [5.1, 5.7]	0.143
**Total Cholesterol (mg/dL)**	147 [128, 168]	147 [126, 166]	153 [133, 181]	0.561
**HDL Cholesterol (mg/dL)**	42 [36, 50]	44 [36, 53]	44 [34, 57]	0.181
**LDL Cholesterol (mg/dL)**	95 [79, 115]	93 [76, 112]	103 [81, 111]	0.339
**Triglycerides (mg/dL)**	79 [56, 109]	70 [50, 102]	93 [65, 115]	**0.017**
**FPG (mg/dL)**	89 [83, 95]	88 [82, 95]	83 [81, 88]	0.214
**2hrPG (mg/dL)**	107 [92, 125]	108 [96, 124]	131 [119, 144]	**<0.001**

Abbreviations: BMI, body mass index; BP, blood pressure; HbA1c, hemoglobin A1c; HDL, high-density lipoprotein; LDL, low-density lipoprotein; FPG, fasting plasma glucose; 2hrPG, 2-hour plasma glucose; Q1, 1st quartile; Q3, 3rd quartile.

1. Median [Q1, Q3]; n (%); shape classifications were undetermined for five participants. Variable-specific missingness existed for BP (n = 21), waist circumference (n = 24), and lipid labs (n = 10).

2. Kruskal-Wallis test (continuous characteristics) or Fisher's exact test (categorical characteristics.

Table S2. Univariate linear regression of standardized glucose FPCs by demographic characteristics (cross-sectional)

Characteristic (Independent Variable)[1]	Dependent Variable											
	Glucose FPC1				Glucose FPC2				Glucose FPC3			
	**Beta**	**95% CI**	**p-value**	**R^2**	**Beta**	**95% CI**	**p-value**	**R^2**	**Beta**	**95% CI**	**p-value**	**R^2**
**Age (Years)**[2]	-0.02	-0.05, 0.01	0.247	0.002	-0.02	-0.06, 0.01	0.154	0.003	-0.05	-0.08, -0.02	**0.002**	0.014
**Sex**[3]				0.006				0.036				0.000
Female	—	—			—	—			—	—		
Male	0.15	0.00, 0.30	0.054		-0.38	-0.53, -0.23	**<0.001**		0.00	-0.16, 0.15	0.951	
**Race**[3]				0.025				0.052				0.003
White	—	—			—	—			—	—		
Black or African American	-0.30	-0.47, -0.13	**<0.001**		0.50	0.34, 0.66	**<0.001**		-0.06	-0.23, 0.11	0.503	
Other/Multiracial	-0.37	-0.66, -0.08	**0.012**		0.26	-0.02, 0.55	0.068		-0.13	-0.42, 0.16	0.363	
Unknown/Not Reported	0.10	-0.34, 0.53	0.656		0.12	-0.31, 0.55	0.571		0.18	-0.26, 0.62	0.434	
**Ethnicity**[3]				0.003				0.000				0.000
Non-Hispanic/Latino	—	—			—	—			—	—		
Hispanic/Latino	0.22	-0.10, 0.54	0.185		0.03	-0.29, 0.36	0.839		-0.03	-0.35, 0.29	0.856	

Abbreviations: FPC, functional principal component; CI, confidence interval.

1. Each independent variable (rows) and FPC (columns) combination represents a separate univariable linear regression model.
2. Betas are interpreted as the standard deviation difference in each glucose FPC per each additional year of age.
3. Betas are interpreted as the standard deviation difference in each glucose FPC associated with each category relative to the reference level.

Table S3. Univariate linear regression of standardized insulin FPCs by demographic characteristics (cross-sectional)

	Dependent Variable											
	**Insulin FPC1**				**Insulin FPC2**				**Insulin FPC3**			
**Characteristic (Independent Variable)**[1]	**Beta**	**95% CI**	**p-value**	**R^2**	**Beta**	**95% CI**	**p-value**	**R^2**	**Beta**	**95% CI**	**p-value**	**R^2**
**Age (Years)**[2]	0.00	-0.03, 0.03	0.908	0.000	0.02	-0.01, 0.05	0.172	0.003	-0.05	-0.08, -0.01	**0.004**	0.012
**Sex**[3]				0.017				0.013				0.008
Female	—	—			—	—			—	—		
Male	-0.26	-0.41, -0.11	**<0.001**		0.23	0.07, 0.38	**0.003**		-0.18	-0.33, -0.03	**0.022**	
**Race**[3]				0.002				0.012				0.012
White	—	—			—	—			—	—		
Black or African American	0.08	-0.08, 0.25	0.327		0.17	0.01, 0.34	**0.042**		0.19	0.02, 0.36	**0.025**	
Other/Multiracial	0.14	-0.15, 0.43	0.339		0.05	-0.23, 0.34	0.712		0.22	-0.07, 0.51	0.132	
Unknown/Not Reported	-0.02	-0.46, 0.42	0.937		-0.37	-0.81, 0.07	0.098		0.38	-0.06, 0.82	0.087	
**Ethnicity**[3]				0.002				0.000				0.000
Non-Hispanic/Latino	—	—			—	—			—	—		
Hispanic/Latino	0.20	-0.12, 0.53	0.220		-0.08	-0.41, 0.24	0.613		-0.09	-0.42, 0.23	0.576	

Abbreviations: FPC, functional principal component; CI, confidence interval.

1. Each independent variable (rows) and FPC (columns) combination represents a separate univariable linear regression model.
2. Betas are interpreted as the standard deviation difference in each insulin FPC per each additional year of age.
3. Betas are interpreted as the standard deviation difference in each insulin FPC associated with each category relative to the reference level.

Table S4. Adjusted linear regression models of standardized glucose FPCs by metabolic health parameters (cross-sectional)

	Dependent Variable											
	Glucose FPC1[1]				Glucose FPC2[1]				Glucose FPC3[1]			
**Independent Variable**	**Beta**	**95% CI**	**p-value**	**Adjusted R^2**	**Beta**	**95% CI**	**p-value**	**Adjusted R^2**	**Beta**	**95% CI**	**p-value**	**Adjusted R^2**
**BMI Percentile**	0.061	0.042, 0.081	**<0.001**	0.076	0.015	-0.004, 0.035	0.128	0.077	-0.003	-0.023, 0.017	0.785	0.007
**Waist Circumference (cm)**	0.010	0.004, 0.015	**0.001**	0.040	0.006	0.000, 0.011	**0.038**	0.079	-0.004	-0.010, 0.002	0.155	0.010
**Systolic BP (per 10 mmHg)**	0.054	-0.011, 0.120	0.106	0.028	-0.036	-0.100, 0.029	0.278	0.070	-0.066	-0.132, -0.001	**0.048**	0.014
**Diastolic BP (per 10 mmHg)**	0.116	0.010, 0.221	**0.031**	0.032	-0.095	-0.198, 0.008	0.070	0.074	-0.057	-0.163, 0.048	0.286	0.010
**HbA1c (%)**	0.733	0.557, 0.908	**<0.001**	0.113	0.060	-0.120, 0.239	0.514	0.075	0.280	0.095, 0.465	**0.003**	0.020
**FPG (per 10 mg/dL)**	0.485	0.414, 0.557	**<0.001**	0.229	-0.297	-0.372, -0.221	**<0.001**	0.151	0.572	0.503, 0.640	**<0.001**	0.293
**2hrPG (per 10 mg/dL)**	0.319	0.301, 0.336	**<0.001**	0.665	0.169	0.143, 0.195	**<0.001**	0.255	0.136	0.107, 0.164	**<0.001**	0.123
**Triglycerides (per 10 mg/dL)**	0.044	0.027, 0.062	**<0.001**	0.059	0.013	-0.005, 0.030	0.147	0.079	-0.003	-0.021, 0.016	0.783	0.007
**HDL Cholesterol (per 10 mg/dL)**	0.122	0.059, 0.185	**<0.001**	0.045	-0.020	-0.082, 0.042	0.525	0.076	0.169	0.106, 0.232	**<0.001**	0.047
**LDL Cholesterol (per 10 mg/dL)**	0.043	0.014, 0.071	**0.004**	0.037	0.015	-0.013, 0.043	0.290	0.077	0.007	-0.022, 0.036	0.644	0.007
**Total Cholesterol (per 10 mg/dL)**	0.052	0.026, 0.077	**<0.001**	0.047	0.006	-0.019, 0.032	0.627	0.076	0.027	0.001, 0.053	**0.043**	0.013

Abbreviations: BMI, body mass index; BP, blood pressure; HbA1c, hemoglobin A1c; HDL, high-density lipoprotein; LDL, low-density lipoprotein; FPG, fasting plasma glucose; 2hrPG, 2-hour plasma glucose.

1. Model adjusted for age, sex, race, and ethnicity. Each independent variable (rows) and FPC (columns) combination represents a distinct regression model. Betas are interpreted as adjusted increase in each glucose FPC per unit (or 10 unit) change in the metabolic health parameter. Variable-specific missingness existed for BP (n = 21), waist circumference (n = 24), and lipid labs (n = 10).

Table S5. Adjusted linear regression models of standardized insulin FPCs by metabolic health parameters (cross-sectional)

	Dependent Variable											
	Insulin FPC1[1]				Insulin FPC2[1]				Insulin FPC3[1]			
**Independent Variable**	**Beta**	**95% CI**	**p-value**	**Adjusted R^2**	**Beta**	**95% CI**	**p-value**	**Adjusted R^2**	**Beta**	**95% CI**	**p-value**	**Adjusted R^2**
**BMI Percentile**	0.106	0.087, 0.124	**<0.001**	0.169	0.015	-0.005, 0.035	0.150	0.023	0.017	-0.003, 0.037	0.103	0.025
**Waist Circumference (cm)**	0.032	0.027, 0.037	**<0.001**	0.191	0.005	-0.001, 0.011	0.077	0.026	0.002	-0.003, 0.008	0.422	0.018
**Systolic BP (per 10 mmHg)**	0.147	0.082, 0.212	**<0.001**	0.040	0.084	0.020, 0.149	**0.011**	0.028	-0.036	-0.101, 0.029	0.275	0.022
**Diastolic BP (per 10 mmHg)**	0.200	0.095, 0.304	**<0.001**	0.031	0.113	0.009, 0.217	**0.033**	0.025	-0.077	-0.181, 0.027	0.148	0.023
**HbA1c (%)**	0.417	0.234, 0.599	**<0.001**	0.039	-0.027	-0.211, 0.158	0.778	0.020	0.150	-0.034, 0.335	0.110	0.025
**FPG (per 10 mg/dL)**	0.135	0.054, 0.215	**0.001**	0.026	0.075	-0.006, 0.155	0.069	0.024	0.079	-0.002, 0.159	0.056	0.026
**2hrPG (per 10 mg/dL)**	0.166	0.139, 0.194	**<0.001**	0.185	-0.152	-0.180, -0.124	**<0.001**	0.166	0.119	0.090, 0.148	**<0.001**	0.110
**Triglycerides (per 10 mg/dL)**	0.091	0.075, 0.108	**<0.001**	0.160	0.009	-0.009, 0.027	0.349	0.021	0.016	-0.002, 0.034	0.078	0.025
**HDL Cholesterol (per 10 mg/dL)**	-0.085	-0.149, -0.021	**0.010**	0.022	-0.041	-0.105, 0.024	0.215	0.022	0.007	-0.057, 0.071	0.825	0.021
**LDL Cholesterol (per 10 mg/dL)**	0.066	0.037, 0.094	**<0.001**	0.041	-0.008	-0.037, 0.021	0.587	0.020	0.019	-0.010, 0.048	0.191	0.023
**Total Cholesterol (per 10 mg/dL)**	0.038	0.012, 0.064	**0.005**	0.024	-0.015	-0.041, 0.011	0.268	0.021	0.020	-0.006, 0.046	0.141	0.024

Abbreviations: FPC, functional principal component; BMI, body mass index; BP, blood pressure; HbA1c, hemoglobin A1c; HDL, high-density lipoprotein; LDL, low-density lipoprotein; FPG, fasting plasma glucose; 2hrPG, 2-hour plasma glucose

1. Model adjusted for age, sex, race, and ethnicity. Each independent variable (rows) and FPC (columns) combination represents a distinct regression model. Betas are interpreted as adjusted increase in each insulin FPC per unit (or 10 unit) change in the metabolic health parameter. Variable-specific missingness existed for BP (n = 21), waist circumference (n = 24), and lipid labs (n = 10).

Table S6. Longitudinal subset participant characteristics at baseline, comparison to cross-sectional sample

Characteristic	Participants in longitudinal subset,[1] N = 193[2]	Participants in cross-sectional sample only, N = 478[2]	p-value[3]
**Age (Years)**	13.3 [11.5, 15.3]	13.5 [11.6, 15.4]	0.229
**Sex**			0.549
Female	108 (56%)	254 (53%)	
Male	85 (44%)	224 (47%)	
**Race**			**0.006**
White	104 (54%)	282 (59%)	
Black or African American	55 (28%)	157 (33%)	
Other/Multiracial	24 (12%)	28 (5.9%)	
Unknown	10 (5.2%)	11 (2.3%)	
**Ethnicity**			0.100
Non-Hispanic/Latino	177 (92%)	455 (95%)	
Hispanic/Latino	16 (8.3%)	23 (4.8%)	
**BMI Percentile**	96.2 [92.1, 98.6]	97.3 (94.6, 98.9)	**0.005**
**FPG (mg/dL)**	83 [79, 90]	91 [85, 97]	**<0.001**
**2hrPG (mg/dL)**	99 [86, 114]	112 [98, 128]	**<0.001**
**HbA1c (%)**	5.2 [5.0, 5.4]	5.4 [5.2, 5.7]	**<0.001**
**Baseline Dysglycemia**			**<0.001**
No	179 (93%)	340 (71%)	
Yes	14 (7.3%)	138 (29%)	

Abbreviations: BMI, body mass index; FPG, fasting plasma glucose; 2hrPG, 2-hour plasma glucose; HbA1c, hemoglobin A1c; Q1, 1st quartile; Q3, 3rd quartile.

1. The Longitudinal subset is defined as participants with baseline and follow up data for whom at least six months passed between visits.
2. Median [Q1, Q3] or n (%).
3. Wilcoxon rank sum test (continuous characteristics) or Fisher's exact test (categorical characteristics).
